# Laparoscopic Artificial Insemination Technique in Small Ruminants—A Procedure Review

**DOI:** 10.3389/fvets.2018.00266

**Published:** 2018-10-23

**Authors:** Swanand R. Sathe

**Affiliations:** Department of Veterinary Clinical Sciences, College of Veterinary Medicine, Iowa State University, Ames, IA, United States

**Keywords:** laparoscopic, AI, ewes, does, insemination, frozen, semen

## Abstract

Laparoscopic artificial insemination (LAI) is an intrauterine method of insemination, especially utilized in the small ruminant species to bypass their unique anatomically tortuous cervix. There are several advantages of LAI that include efficient use of processed semen leading to higher pregnancy rates. Success of LAI programs depends on proper implementation of estrus synchronization programs, patient selection and thorough knowledge of the reproductive physiology. In addition, proper equipment and surgical expertise help in reducing patient morbidity and mortality rates. LAI can be associated with several complications as a result of inadequate patient preparation, poor technique or equipment failure. Hence, a thorough planning is essential to carry out the procedure safely and with consistent success rates. Addition of LAI to a small ruminant/food animal practice can be quite profitable and professionally fulfilling, as long as an appropriate investment in equipment and adequate training of veterinarians and technical staff is implemented. Though the technique for performing LAI has been mentioned briefly through various research articles, this article serves as the first comprehensive review of the technique, equipment used, associated complications and useful practical tips that could serve as a guideline for clinicians interested in adding this service to their practice. The article also contains some novel research supported ideas to provide laparoscopic insufflation that have been recently developed.

## Introduction and background

Assisted reproductive technologies (ART) are utilized in animal reproduction to promote efficient use of germplasm for improvement of genetic value of companion and production animals. Artificial insemination (AI) is one such ART that has revolutionized the cattle breeding industry within the past few decades. With development of more efficient methods of semen cryopreservation and estrus synchronization, it is now possible to obtain higher pregnancy rates even with poor quality frozen semen via artificial insemination. Unlike cattle though, the small ruminant semen industry in the US is unorganized and semen testing and cryopreservation parameters are not rigorously implemented. There is a growing demand for use of frozen and processed semen especially in the show lamb industry, where owners are willing to pay a premium to introduce newer genetics in their flocks. However, ewes/does bred with frozen or processed semen typically have a lower pregnancy rate when bred via conventional methods such as vaginal (VAI) and trans-cervical artificial insemination (TCAI) techniques. This is due to the long and tortuous nature of the cervix in small ruminants and the presence of 4–7 cervical rings. These cervical rings point caudally into the lumen thus providing a physical barrier (1–5) to attempts at passing an insemination pipette during TCAI. There is ample evidence that pregnancy (6) and lambing rates (7) improve as the depth of semen deposition in the cervix increases. The degree of penetration of the insemination pipette through the cervix depends on the breed, age and stage of the estrous cycle especially in ewes (8). Deeper semen deposition results in greater numbers of motile spermatozoa available for fertilization thus leading to higher pregnancy rates (9).

Laparoscopic artificial insemination is an advanced assisted reproductive technique that enables such deep intrauterine deposition of semen and helps bypass the physical barriers of the caudal reproductive tract in small ruminants. Pregnancy rates using frozen semen deposited intra-uterine via laparoscopy have yielded higher pregnancy rates (60–80%) consistently when compared to vaginal and TCAI methods (10–12). In addition to higher pregnancy rates, it is possible to use lower concentrations of spermatozoa per breeding thus leading to more number of animals bred per ejaculate. The average dose required for breeding a ewe using frozen semen can be as less as 20–25 million live spermatozoa, when compared to higher doses required via the vaginal (400 million live spermatozoa) and trans-cervical route (100–200 million live spermatozoa) (13). Thus, a single entire frozen ejaculate can be used to inseminate as many as 50 to 100 ewes, thus leading to a more efficient use of semen. The main disadvantage of providing LAI service to producers has been the high equipment cost and the relatively steep curve of surgical expertise required to perform to perform the procedure safely. However, with the availability of newer and more portable laparoscopy equipment, it is now possible to offer these services cost-effectively at a hospital and field setting. The following paper will discuss the LAI procedure in detail with emphasis on surgical techniques, equipment needed, and useful practice tips based on the authors experience at the Theriogenology Service at Iowa State University, College of Veterinary Medicine.

## Materials required

Most equipment and associated costs quoted here are from the manufacturer Karl Storz®, Germany, though there are other manufacturers available in the market as well.

a) Trocars and Cannulas—There are two common sizes available that suit the purpose (i) 10 mm and (ii) 5 mm. Estimated cost $500.

It is preferable to use the smaller size as there are several advantages: (i) Enables creating smaller incisions and ports, (ii) Lesser chances of abdominal perforation due to lesser effort required during insertion (iii) Lesser wear and tear of instruments, (iv) No need for trocar adaptors for AI guns (v) Enables use of smaller rigid 5 mm telescopes (laparoscopes).

b) Telescope/Laparoscope (available options are 0 and 30°): We prefer using a 0°scope, but a 30° oblique telescope can also be used effectively. Estimated cost $7,000 (new)—$3,500 (refurbished).
c) Light Source with halogen/xenon bulbs and cables: Approximate cost around $7,000.

There are handheld portable endoscope light sources available now which are better suited for field work and just as effective costing around $500.

d) Video camera and screen (optional, since many practitioners in a field setting prefer looking through the lens of the laparoscope instead of using a camera and screen). Approximate cost $3,500–4,000.

A new mobile video-endoscopy unit available from Karl Storz® known as the Tele Pack Vet X Led® is multifunctional and contains a camera, light source; air insufflation unit and image capture capabilities. This unit is light, portable and can be carried out to the field in a carry case (provided). It can also be adapted for concurrent use in small animal and equine clinical practice. Approximate cost $25,000.

e) Air insufflation unit—A medical grade air insufflation unit with carbon dioxide tank can be used to ensure an ideal abdominal pressure. However, the equipment is expensive and cumbersome to move around especially when performing this procedure at a field level. Also, the rate of insufflation is relatively slower leading to increased surgical time and increased duration of the patient in a Trendelenburg position. Instead of a traditional air insufflation unit, a regulator can be attached directly to the CO_2_ tank which enables faster insufflation. The disadvantage is that the degree of insufflation is subjective and based on abdominal percussion and degree of distention. Yet another alternative is to use a commercial vacuum pump system (e.g., GAST® vacuum pump) with an attached inline filter. The advantage of using such filter systems is that besides CO_2_, other alternative gases such as medical grade air or room air can also be used for insufflation. Disadvantages of using room air are that it is risky to use in a dusty environment due to the danger of causing peritonitis. A recent research study (14) has shown that there is no danger of hypoxia or infections in using medical grade air vs. CO_2_. Nor were there any differences in pregnancy rates observed between the two groups. In a hospital setting we prefer to use the vacuum pump and insufflate with room air. We have performed more than 400 surgical procedures using room air, without any reports of complications such as peritonitis or septicemia. There were no differences found in pregnancies rates with use of room/medical air as compared to CO_2_.

Medical grade air insufflation unit: $8,000, CO_2_ tank with regulator: $250, Commercial vacuum pump (e.g., GAST® Mfg.): $500.

f) Laparotomy surgical pack: We recommend having ready access to a laparotomy surgical pack, for the purpose of isolating and suturing any subcutaneous bleeding vessels after the LAI procedure or for performing an emergency laparotomy in case of an abdominal organ perforation.
g) Laparoscopy AI cradle: These are specialized cradles that can be tilted almost up to 90° to position animals in a Trendelenburg position. We recommend buying cradles with aluminum frames as they are sturdy yet light to transport in case of field work (Sydell® Iowa, Minitube® USA etc.). Approximate cost: $1,500.
h) Semen Processing:

(i) AI guns and sheaths: Transcap with guide (IMV®, France): $550; Aspics for semen straws: $70/25 pcs; Robertson's AI gun with sheaths (Minitube® USA): $500.

(ii) Semen processing equipment: Semen tube holders, semen straw thawing equipment, slide warmer, portable microscope, 0.25 cc straws, straw cutters etc. (Total cost around $3,000 including the microscope).

## Laparoscopic AI procedure

### Selection of patients

LAI though minimally invasive is still a surgical procedure nonetheless and hence young, healthy ewes/does in appropriate body condition scores (BCS's) are ideal candidates for the surgery. It has been shown that an optimum BCS results in higher ovulation as well as pregnancy rates (15, 16). Fat/obese animals not only prove to be a surgical risk, but also may not respond appropriately to synchronization protocols (AI or Embryo transfer), thus increasing the net surgical procedure time. A decrease in embryonic viability and subsequently lower pregnancy rates have been observed in animals maintained on a body score of below 2 and above 4 (17). The proposed mechanism behind pathogenesis in overly conditioned animals has been attributed to lower progesterone levels possibly because of increased liver blood flow, leading to increased clearance of the hormone from circulation (18). The preparation of animals for estrus synchronization and LAI begins several weeks prior to actual date of the procedure. This involves vaccinations, deworming and increasing the plane of nutrition (flushing) to achieve an ideal BCS. However, in a recent report (19) the pregnancy rates declined in ewes handled 4–6 weeks prior to LAI for various managemental procedures such as deworming, vaccinations and feet trimming. A possible explanation to this decreased pregnancy rate was attributed to the stress experienced during such procedures. Hence, special care in terms of minimizing stress should be undertaken when handling animals for routine preventive managemental procedures. In addition, attention should be paid to detect the presence of systemic diseases in the flock such as infections of the respiratory system which increases surgical risk to the affected animal. The estrus synchronization protocols are sent out several weeks prior to the day of the procedure. A thorough knowledge of reproductive physiology, seasonal and breed variations, appropriate duration of protocols and drug dosages are essential for adequate response from patients. Shorter protocols can be designed for animals that are cycling and during breeding season, whereas out of season protocols tend to be longer in order to prime the inactive reproductive tract for estrus. Older animals that have undergone surgical embryo transfers or multiple LAI procedures in the past can have extensive adhesions of the omentum to the parietal peritoneum thus forming a curtain/barrier or, in some cases adhesions to the reproductive tract. This can lead to difficulties in visualization of the reproductive tract and increases the duration of surgery or the need to abandon the procedure halfway. Based on the reproductive history, animals suspected of being pregnant should be checked via trans-abdominal ultrasound examination before initiating the synchronization protocols. Since the LAI procedure involves restraining animals in a Trendelenburg position, it is recommended to keep the animals off-feed for at least 16–20 h and off-water for at least 12 h to prevent abdominal fill and minimize chances of regurgitation and aspiration.

### Sedation and premedication

Light sedation is usually recommended since the total duration of the procedure (preparation and surgery) takes roughly about 10–15 min only. The goal is to have the patient stand up on their feet after they are loaded off the cradle and start searching for food.

Two classes of drugs that we recommend for sedation are alpha-2-agonists (Inj. Xylazine@ 0.05–0.1 mg/kg of the 20 mg/ml large animal formulation I.V. or I.M.) and tranquilizers (Acepromazine@ 0.05–0.1 mg/kg I.V. or I.M.). Opioids such as Butorphanol (0.05–0.2 mg/kg IV, IM, SQ) may also be used 5 to 10 min before handling animals for restraint. The advantage is that they afford some degree of analgesia, but personal experience has shown that it also leads to lesser degree of sedation than alpha-2-agonists, causing more struggling among patients when they are restrained in a Trendelenburg position. Other medication such as anti-inflammatories (Flunixin meglumine@ 1.1 mg/kg I.V.) and in some cases long acting antibiotics (Ceftiofur crystalline free acid or Long acting Oxytetracycline- extra label usage) can also be administered during restraint and surgical preparation. Local analgesia in form of Lidocaine hydrochloride is administered at each proposed incision site (2 ml of 2% Lidocaine hydrochloride subcutaneously) and each site is scored with a hypodermic needle to mark the area for future reference. An appropriate withdrawal time for meat and milk should be relayed to the producer when using any of the above drugs (www.farad.org).

### Pre-surgical preparation

**Figure 1:**
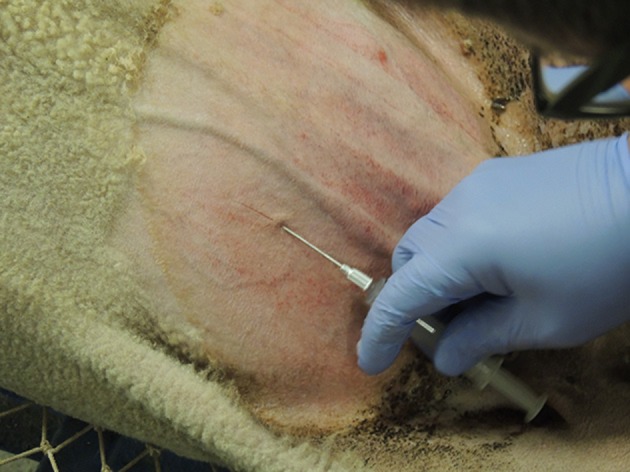
Surgical sites are located cranial to the udder and medial or lateral to the mammary/superficial epigastric veins. Local anesthetic is infiltrated, and the sites are scored to identify them prior to the surgical procedure. Left of the image is cranial.

The patient (ewe/doe) is restrained and sedated with the appropriate sedative agent and observed for clinical effects. Once adequate sedation is confirmed, the animal is then lifted and restrained on a special, custom made laparoscopic AI cradle. The fore- and hind-feet (at level of hocks) are restrained securely, a face mask or a towel is used to cover the eyes and the animal moved to the surgical-prep station. The wool on the ventral abdomen is clipped from the level of the mammary glands and extending cranially up to the umbilicus. The area is surgically scrubbed with 2% Chlorhexidine scrub alternating with 70% isopropyl alcohol. Special attention is to be paid to the inguinal gutters as they accumulate loose dirt, dried feces, natural sebaceous secretion, and which tend to contaminate the surgical site when the animal is suspended in a Trendelenburg position. Two sites about a hands width cranial to mammary glands and adjacent to the left and right mammary/superficial epigastric veins are identified. The site can be medial or lateral to the veins and the choice is dependent on the preference of the surgeon and size of the patient. For larger patients (>120 lbs.), we recommend a more medial approach since the abdomen is wide and tends to get wider when insufflated with CO_2_/air. This prevents the laparoscopic instruments from reaching the reproductive tract or aligning with each other during the insemination process. On smaller patients (< 120 lbs.), a more lateral approach is recommended to afford adequate insufflation and avoid more medially placed abdominal organs. The surgical sites thus selected are superficially scored with a 20 G-1-inch needle, and 2 ml. of 2% Lidocaine hydrochloride infiltrated in the subcutaneous tissues and musculature (Figure 1). The skin scoring is performed to identify the surgical sites during surgical procedure, since the local anesthetic tends to dissipate quickly. A final surgical scrub is performed before wheeling the patient to the surgical station.

### Surgical procedure

**Figure 2:**
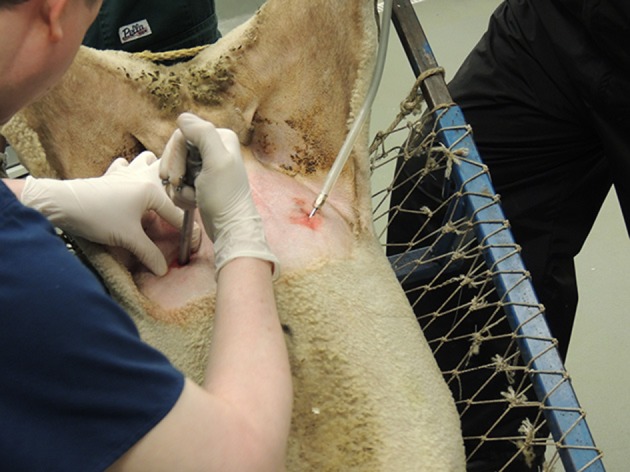
A 5 or 10 mm trocar and cannula pointing laterally, are inserted through the near incision into the abdominal cavity with a firm pressure and the trocar withdrawn to be replaced with a laparoscope.

**Figure 3:**
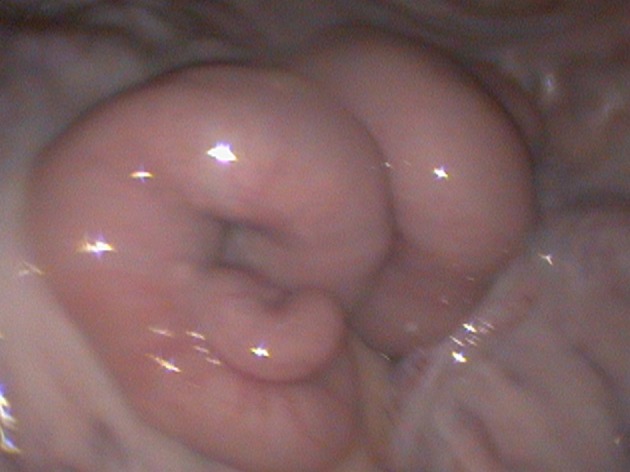
Intra-abdominal view of the reproductive tract. The uterine horns have a distinct tone and color under the influence of estrogen. This type of appearance is classified as Grade 2.

**Figure 4:**
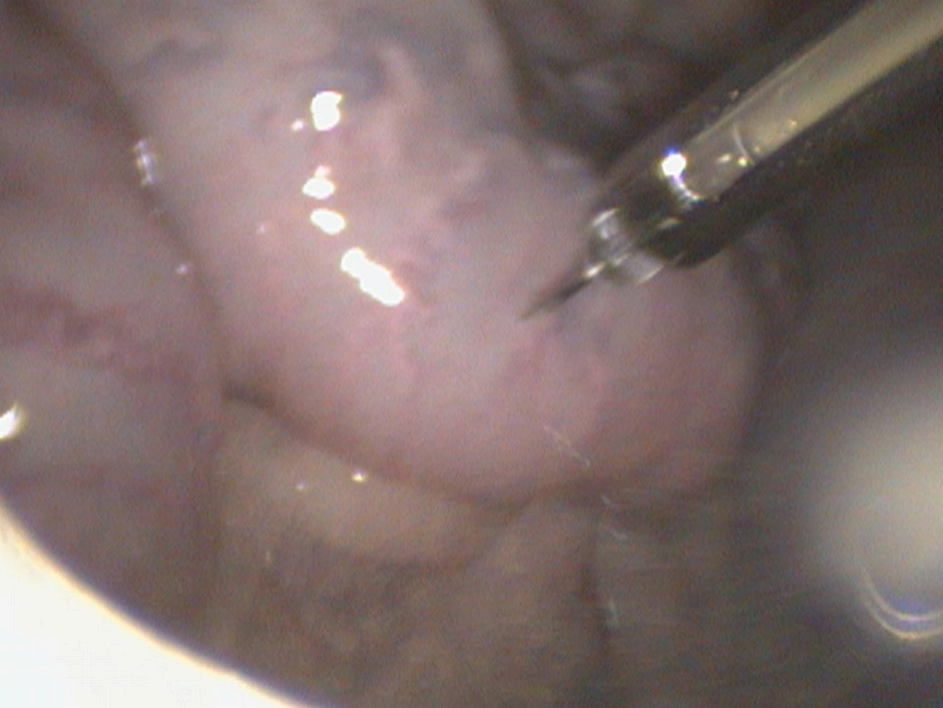
A laparoscopic AI gun and needle apparatus are used to inject semen intra-uterine at the level of the mid-horn along the greater curvature.

The LAI cradle is elevated up to 45°, to position the ewe in a Trendelenburg position. A no. 11 scalpel blade is used to create 0.5 inch incisions through the skin and fascia up to the level of muscle over the proposed pre-scored incision sites. A 1.5-inch blunt teat cannula attached to a sterile flexible insufflation hose is inserted pointing laterally through the muscle layers and intra-abdominally via the incision farther from the surgeon with a firm, calculated push. Air insufflation is carried out till the ventral abdomen feels adequately tense. With practice it is possible to carry out LAI with lesser amounts of air safely. The advantage of insufflating lesser amount of air is to reduce the degree of hypoxia to the patient while in Trendelenburg position. A 5 mm trocar and cannula are inserted in the abdomen through the near incision with calculated pressure (Figure 2), the trocar withdrawn, and a 5 mm Telescope/Laparoscope inserted via the cannula to visualize the interior of the caudal abdomen. The reproductive tract (uterine body and horns) are usually located ventral (from a surgeons' point of view) to the urinary bladder. In animals that have responded adequately to estrus synchronization protocols, a distinct tone and hyperemia can be identified affecting the reproductive tract. The tract appears pale to dark pink and responds by curling when it is touched by the laparoscopic instruments (Figure 3). The location of the tract and its relation to surrounding structures is noted to ascertain the ease with which an intra-uterine injection can be performed safely. On some occasions, a distended bladder can hide the reproductive tract partially or completely. Decompression of the bladder during premedication and surgical preparation is strongly recommended to prevent this from happening. The caudal sac of the rumen, distended cecum or loops of small intestine can sometimes prevent visualization of the reproductive tract. This can be minimized by keeping the patient off feed and water as recommended. Once the reproductive tract is visualized, another similar sized trocar and cannula is inserted adjacent to the teat cannula. A loaded laparoscopic AI gun carrying a 0.25 cc semen straw with an external sheath (IMV®, France) is then inserted through this port and aligned opposite the greater curvature of uterine horns under laparoscopic guidance. The external sleeve/guide can be used to manipulate the uterine horns from underneath overlying structures such as the bladder or omentum to the desired angle. The Aspic and needle apparatus is then exposed keeping them as close to the uterine horns as possible and with a quick jab the needle is seated at the level of the mid-horn. Semen can be injected either in one or both uterine horn as per the operator's preference (Figure 4). Where a single 0.25 cc straw is to be utilized per breeding we prefer injecting both horns with half the straw of semen. However, when a 0.5 cc straw (divided in two aspic guns) is available for use per insemination, an entire gun can be injected per horn. Care is to be taken to ensure proper depth of the needle placement while inseminating to prevent semen leaking in the abdomen. There are no reported advantages of injecting both horns, and the entire dose can be deposited in one horn only, resulting in similar pregnancy rates (20). However, when performing LAI procedures in animals superovulated for embryo recovery, it might be beneficial to inject both horns so that adequate numbers of spermatozoa are available on both sides for migration and fertilization in presence of increased intrauterine mucus due to higher circulating estrogen levels. In addition, injecting both horns also serves as insurance if there is leakage of semen from or incomplete insertion of the lumen at one injection site.

Once the uterine horns are injected, a quick assessment is made to ascertain that there is no excessive bleeding or uterine horn lacerations. The laparoscope and the AI gun are withdrawn from the cannulas. The spring loaded or side valves are decompressed to deflate the abdomen and the animal is lowered to a horizontal plane after removal of all instruments from the abdominal ports.

The skin incisions are closed with the help of non-absorbable suture material (Prolene® 2-0, Ethicon®, USA) in a cruciate suture pattern after ensuring that there is no excessive bleeding. On occasion, subcutaneous branches of the mammary/superficial epigastric veins may bleed excessively. The bleeding vessels are clamped and ligated individually and then the skin incisions closed in an interrupted suture pattern. Skin staplers can also be used and are faster to apply but can be costlier than suture material. We prefer to cover the abdominal incisions with a water resistant antibiotic free aluminum based wound spray (Aluspray®, Neogen® Animal Safety, USA). We also prefer administering a reversal agent in case alpha-2-agonists were used for sedation (e.g., Tolazoline, Yohimbine) as soon as the animal returns to a horizontal plane. Most animals stand up immediately or at least assume a sternal recumbency after being placed on the ground. In cases of prolonged lateral recumbency it is advised to monitor the animal's vital signs closely and prop the animal in a sternal position to avoid aspiration pneumonia.

### Complications arising during laparoscopic AI procedure

As with any surgery, there are numerous complications that can arise during the LAI procedure.

#### Complication arising during insemination of systemically unhealthy animals

One of the major concerns when carrying out LAI is patient mortality due to underlying systemic disease conditions such as respiratory tract infections. A thorough history and physical examination of preferably each animal (when dealing with small groups) is crucial in order to avoid identifying these unhealthy patients. Each animal should be thoroughly observed and checked for signs of coughing and wheezing, especially evident when handling or segregating animals in different groups or during premedication. Despite taking due care, there may be animals that are missed, raising the percent morbidity and mortality rates. Minimizing the surgical procedure time greatly helps in reducing chances of such complications, and is largely dependent on surgical speed and efficient teamwork which is gained through practice. Judging the tone and color of the reproductive tract greatly helps in assessing whether the patients have responded adequately to estrus synchronization protocols. For patients that have poorly responded or not responded at all (flaccid, pale tract) to the hormonal protocols, a decision can thus be reached quickly to abandon the insemination process early enough during surgery. It is always important to constantly monitor vital parameters and signs for regurgitation from the nose/mouth to prevent aspiration pneumonia. Flow-by oxygen via face mask can also help in lessening the degree of hypoxia experienced during the procedure. In animals that are obese or metabolically compensated, we advise the owner against performing the procedure, or decreasing angle of elevation from 45 to 30° after insufflating the abdomen. This reduces pressure on the diaphragm and lessens the degree of hypoxia experienced during the surgical procedure.

#### Rupture/puncture of abdominal viscera

**Figure 5:**
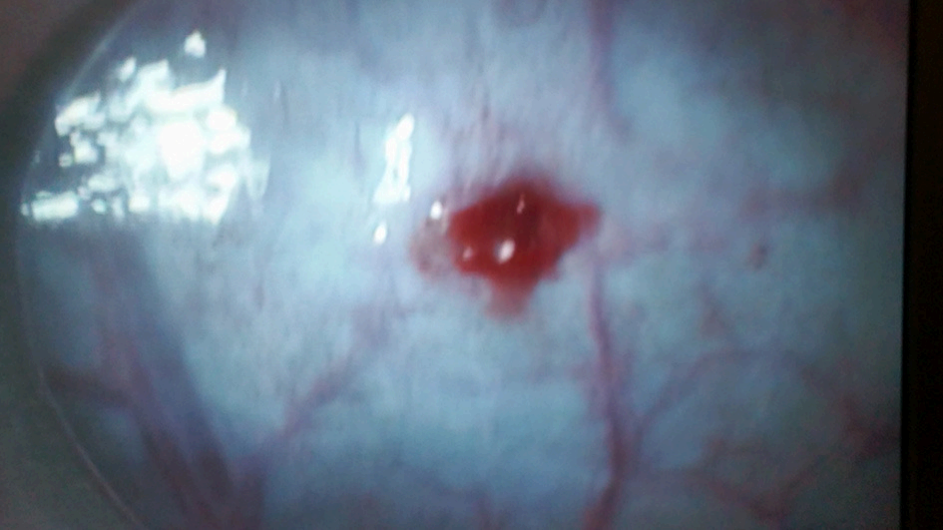
Abdominal organs such as the rumen and the cecum are frequently in danger of rupture during trocar placement especially if the animals are inadequately fasted. The following figure shows a cecal rupture due to insufficient duration of feed and water withholding.

The abdominal organs in danger of being punctured are the urinary bladder in the caudal abdomen, small intestinal loops and cecum in the mid-abdomen and caudal sac of the rumen in the cranial abdomen (Figure 5). To minimize the risk of perforation there are a few things to be kept in mind before and during the surgical procedure:

- Ensure that the animals are adequately fasted and kept off-water.
- Ensure that the urinary bladder is emptied/decompressed to minimize chances of perforation
- Proper and adequate insufflation of the abdominal cavity.
- Proper placement of the trocars: The surgical sites for trocar placement (as described before) should be meticulously selected. Too cranial a site can result in perforation of the caudal sac of the rumen. Too median a placement can result in mammary vein punctures, urinary bladder ruptures, cecal, and small bowel perforations. While placing the trocars through the proposed surgical sites it is also important to angle them laterally. Using smaller trocars (5 mm) also enables for easier insertions through the body wall. The larger 10 mm trocars can create a considerable drag leading to greater force required for insertion and subsequently greater risk of perforation of abdominal organs.

**Figure 6:**
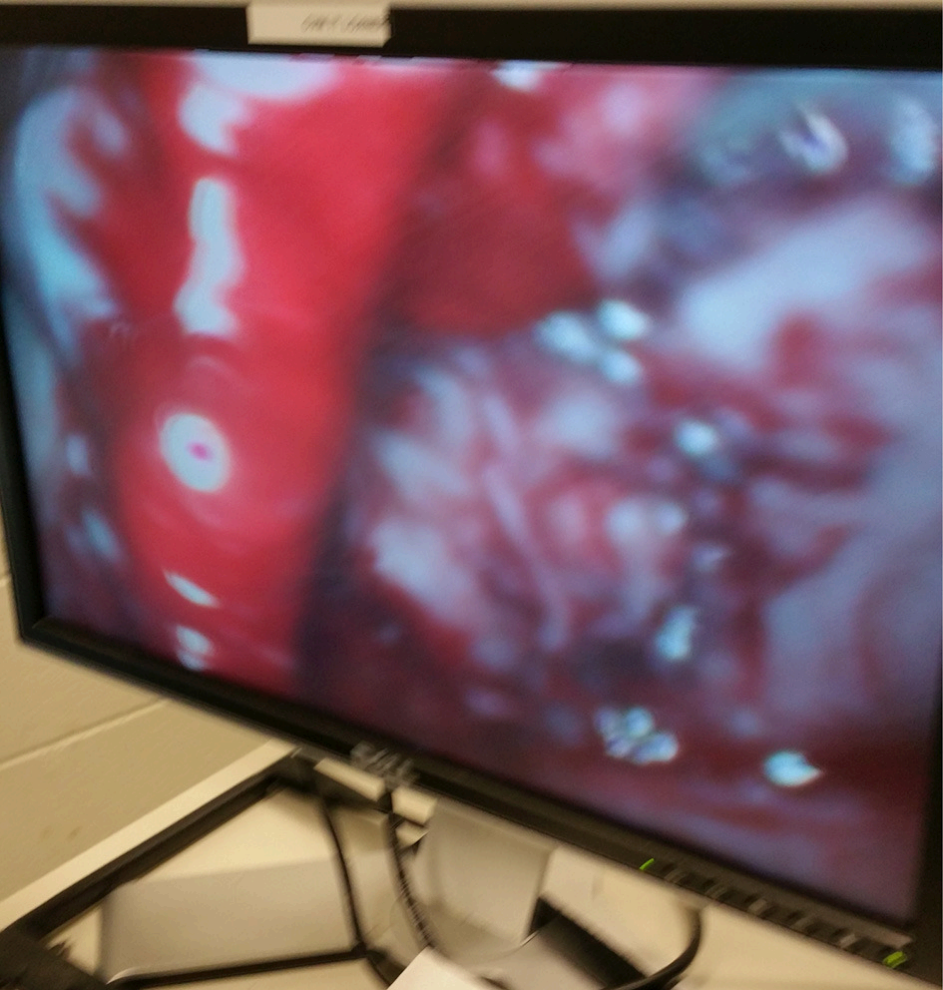
Iatrogenic bladder rupture during trocar insertion due to inadequate decompression prior to surgery.

**Figure 7:**
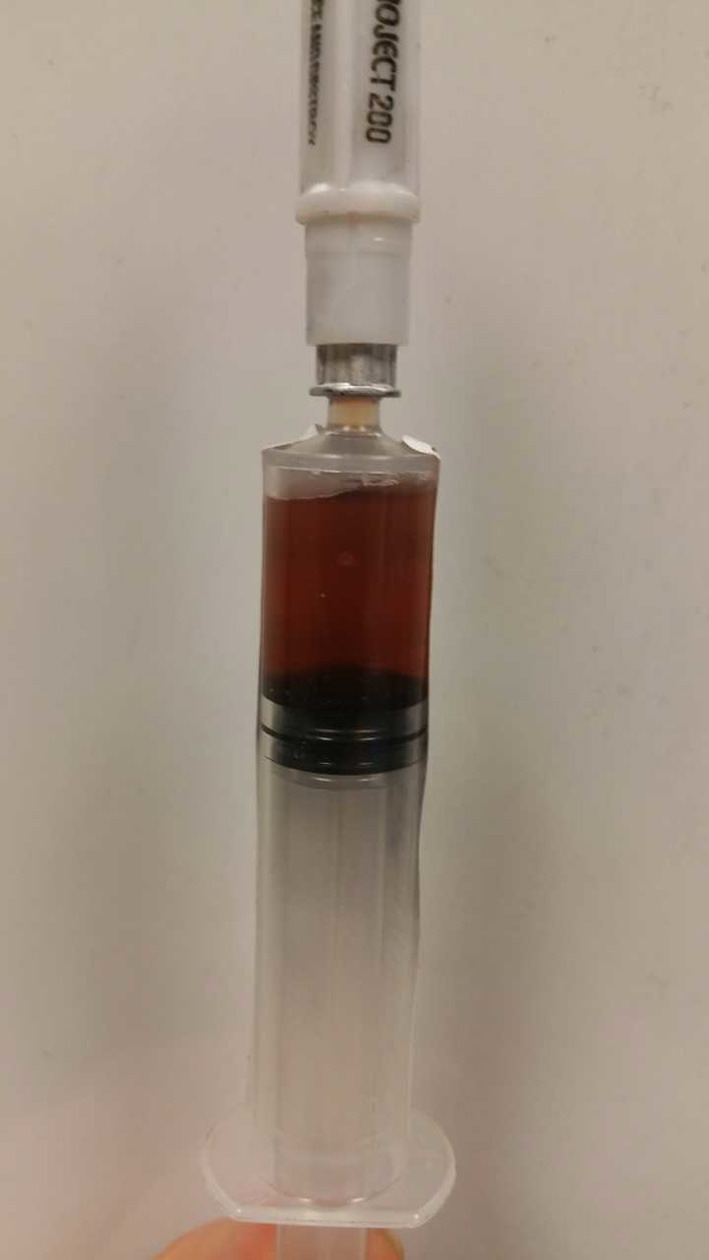
An abdominocentesis post-iatrogenic bladder rupture revealing tan colored fluid consistent with urine.

One of the early signs of a possible gastrointestinal tract perforation is methane like odor evident from the cannula immediately after removal of the trocar. The trocar may have greenish-yellow fecal contents stuck on its end and along the shaft. Bladder ruptures should be suspected with presence of a sudden increase in blood tinged fluid evident in the abdominal cavity (Figures 6, 7). Most ruptures can be confirmed on direct visualization of the organ. However, on some occasions the sudden deflation of a distended viscus can change shape and orientation of the affected organ thus preventing confirmation by direct visualization. On rare occasions, rupture of a major blood vessel such as the aorta or its branches can lead to excessive bleeding and sudden death.

#### Subcutaneous emphysema

In larger animals having a broad abdominal girth there is risk of subcutaneous emphysema developing if the trocars are placed too laterally. This disrupts the normal tissue planes and increases risk of the trocar getting caught between different layers of the abdominal wall, making it difficult to enter the abdominal cavity. In cases of subcutaneous emphysema, the air insufflation needs to be stopped immediately and the leaked air gently massaged out of the incision site. If addressed early, the same site can be utilized for trocar placement. But if a significant amount of subcutaneous air is present, it is best to select another surgical site after suturing the earlier port.

#### Abscess formation/peritonitis/sepsis

A small percentage of animals in the flock may develop peritonitis, external or internal abscesses, sepsis and death. This usually happens when contaminated instruments are used without cleaning them between animals. We recommend dipping and thoroughly wiping off the instruments using 70% alcohol or dilute 2% Chlorhexidine solution, after each surgery and before they can be used for another animal.

#### Hematoma/subcutaneous bleeding

Due to the proximity of surgical site to branches of the mammary/superficial epigastric veins, it is possible to accidently incise a collateral branch leading to excessive subcutaneous bleeding. The blood can leak into abdominal cavity coating laparoscopic instruments and blurring the visual field. Presence of blood can also lead to formation of intra-abdominal adhesions in future. Post-surgery, these vessels can continue bleeding in the subcutaneous tissue leading to formation of a localized hematoma. Since the clotted blood and fibrin are a good media for bacteria to thrive, this can lead to formation of an abscess. After the abdomen is deflated and laparoscopic instruments withdrawn, the surgical sites should be meticulously checked for presence of small bleeding vessels. These should be ligated before suturing/stapling the incision sites.

#### Intra-abdominal adhesions

**Figure 8:**
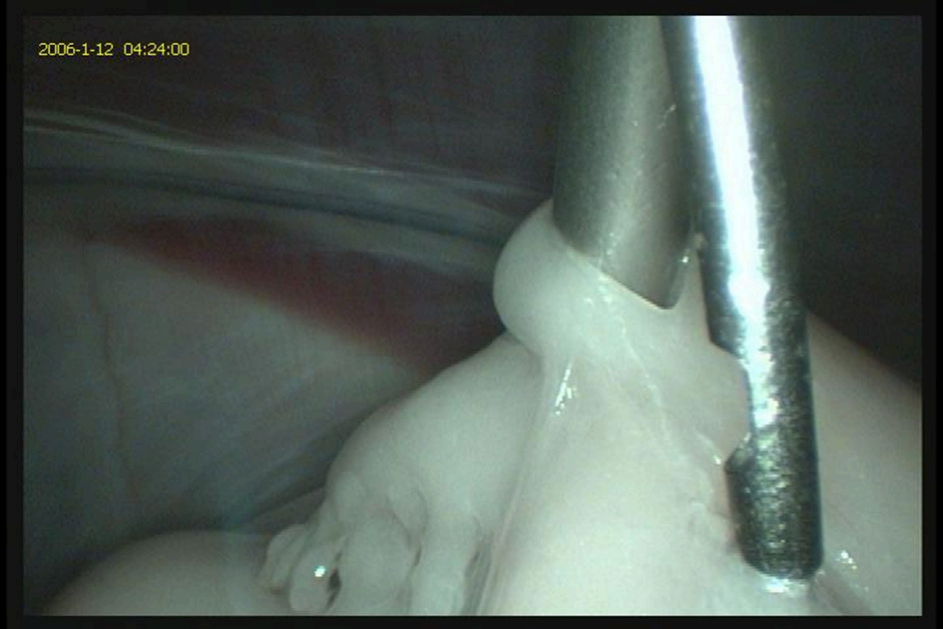
The omentum and omental fat can sometimes get entangled with the laparoscopic instruments due to insufficient insufflation. This can prevent visualization of the reproductive tract and other abdominal contents.

In animals having undergone LAI procedures or surgical embryo flushes in the past, intra-abdominal adhesions of the omentum to the body wall can form. These result in a partial or complete division of the abdominal cavity thus making it difficult to align laparoscopy instruments through the two opposite ports. Efforts to maneuver the instruments around the adhesions or sometimes injecting through the omental barrier are the only options available. On occasions creating three abdominal ports or creating ports on the same side of the body can help circumvent around these adhesions. Sometimes incomplete insufflation can lead to the omentum getting caught on end of the laparoscopic instruments (Figure 8). Adequate insufflation of the abdominal cavity usually results in the omentum falling off instruments affording a clear view of the abdomen.

#### AI gun failure

Sometimes the AI guns may get stuck due to faulty “O” rings or due to faulty batch of semen straws. On occasion, because of “O” ring failure, the injecting straw can disengage and fall in to abdomen (in case of Aspic straws, IMV®) increasing the risk of abdominal organ perforation due to the exposed injection needle. Hence, a laparoscopic grasping forceps should be included in the LAI surgery pack to address such complications. It is always a good idea to load an AI gun with a mock straw filled with saline before start of the procedure to ensure that all parts of the instrument are working well.

#### Inability to seat the injection needle in the uterine lumen

**Figure 9:**
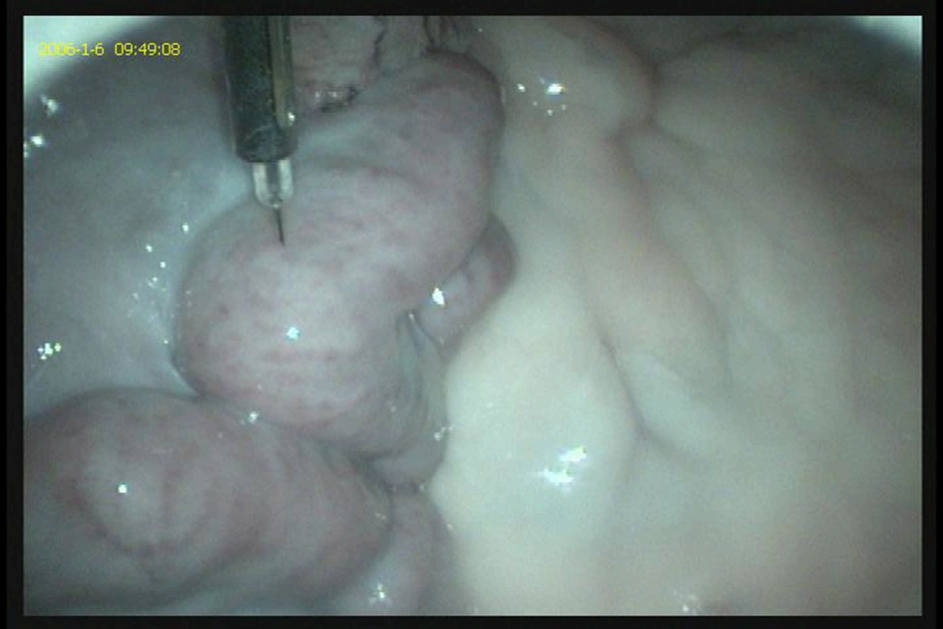
Grade 1 uterine tone and color showing a relatively pale uterus with minimal tone.

In cases where the animals fail to respond adequately to synchronization protocols, the AI gun needle may slip out of the uterine musculature because of the lack of uterine tone. Owners should be informed about such lack of response, the AI process stopped and the animal resynchronized. We document the color and tone observed for each animal during the surgical process. This provides us and the owner a valuable feedback when observing for pregnancy rates and infertility problems in the flock. The uterine tone is graded as: Grade 0—No response; Grade 1- medium degree of response (Figure 9); Grade 2—adequate/good response (Figure 3). The color can vary from pale to different shades of pink depending on stage of estrus. There has been no difference observed in pregnancy rates between Grade 1 and Grade 2 uterine tone and color based on a recent retrospective study conducted by the author and colleagues (unpublished data). The uterine horn increases in thickness during superovulation protocols as compared to conventional AI protocols, and hence a marginally longer aspic injection needle should be used to ensure the proper intraluminal deposition of semen.

#### Uterine bleeding

**Figure 10:**
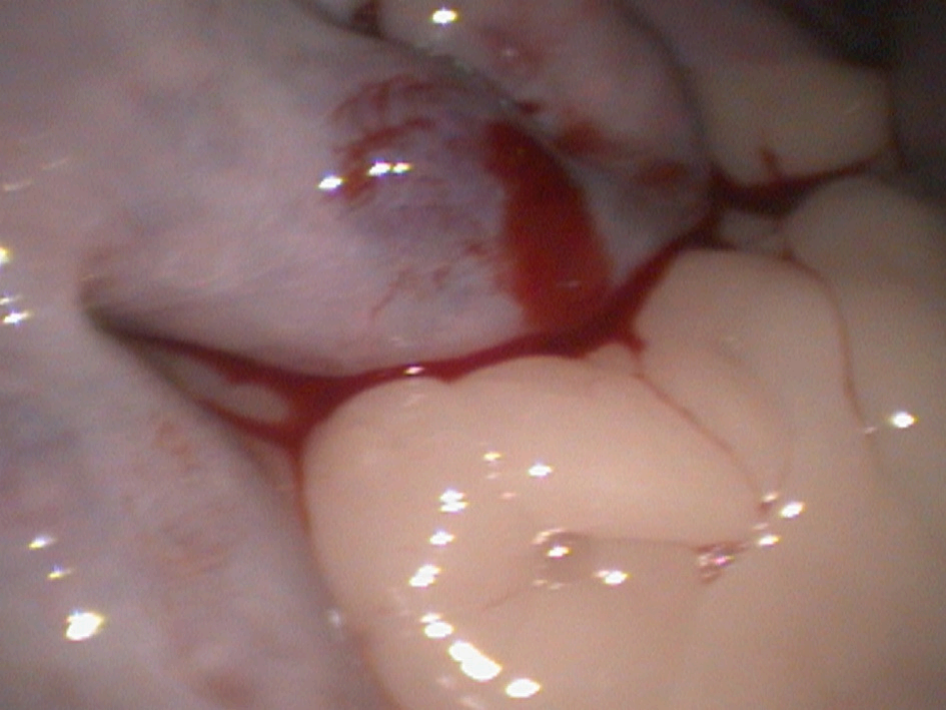
Accidental injection of semen along the medial or lesser uterine curvature can cause bleeding and hematoma formation due to the extensive vasculature in the broad ligament.

The uterine tract in estrus has increased blood supply; hence a little oozing from the injection sites is expected. To minimize extensive bleeding or prevent uterine vessel lacerations, it is advisable to inject along the greater curvature of the uterine horns. The lesser curvature which is close to the broad ligament has a rich vasculature and is prone to bleed extensively if injected (Figure 10).

The most common complications observed are hypoxia in over conditioned animals, subcutaneous bleeding, omental adhesions from prior surgeries and localized hematomas. On rare occasions, we have observed perforations of the gastrointestinal organs (in unfasted animals) and the urinary bladder (21). These usually occur during trocar placements, thus highlighting the importance of feed/water withdrawal and bladder decompression prior to conducting the procedure.

## Conclusions

LAI involves a substantial investment in form of buying equipment and training for the procedure. The success of LAI program requires a good team work and coordination. A trained veterinarian performs the LAI, a trained technician/assistant handles semen processing and other personnel take part in handling, sedating and prepping animals for surgery. On occasion, producers and owners can be trained adequately to prepare animals for the surgery. It takes a few attempts for the team to get used to the flow of the procedure. However, once the operator and the team become proficient with the process, it is possible to inseminate as many as 100–200 animals per day. Addition of LAI to your small ruminant private practice can thus be an economically feasible option that can turn profitable quickly, once producers see an increased pregnancy rate even with use of processed semen.

## Ethics statement

The examples of complications and pictures provided in the manuscript were sourced form the patient pool presented at the Veterinary Teaching Hospital at Iowa State University. All necessary client consents for the procedure as well as use of clinical data for teaching and publishing purposes has been obtained from owners of these animals as a part of routine hospital admission protocol.

## Author contributions

The author confirms being the sole contributor of this work and has approved it for publication.

### Conflict of interest statement

The author declares that the research was conducted in the absence of any commercial or financial relationships that could be construed as a potential conflict of interest.
